# Association between temporal muscle morphology based on MRI and muscle mass and strength in healthy young adults: a cross-sectional study

**DOI:** 10.1186/s12891-026-09713-0

**Published:** 2026-03-07

**Authors:** Xiaojia Wu, Hongwu Yang, Wanting Zheng, Haiqi Hu, Zhanpeng Zheng, Ruyao Zhuang, Ruibin Huang

**Affiliations:** 1 Department of Radiology, The First Affiliated Hospital of Shantou University Medical College, Shantou, Guangdong 515041 China; 2 Shantou University Medical College, Shantou, Guangdong China; 3 Dafeng Hospital, Shantou, Guangdong China

**Keywords:** Temporal muscle thickness, Temporal muscle area, Systemic muscle, Healthy cohorts, Magnetic Resonance Imaging

## Abstract

**Background:**

Temporal muscle morphology, easily visible on cranial magnetic resonance imaging (MRI), is increasingly recognized as a noninvasive biomarker of systemic muscle health. This study examines the association between temporal muscle thickness (TMT) and temporal muscle area (TMA) with skeletal muscle mass, strength, and physical performance in healthy young adults.

**Methods:**

This prospective cross-sectional study involved 61 healthy adults (29 men and 32 women; mean age: 21.7 ± 5.4 years). Participants underwent high-resolution 3T cranial and lumbar magnetic resonance imaging (MRI) between September and October 2025. TMT, TMA and lumbar skeletal muscle cross-sectional area at the third lumbar vertebra (L3-CSA) were systematically quantified. In addition, grip strength, gait speed, and anthropometric measurements were assessed to evaluate functional and morphological aspects of muscle condition. Correlations between temporal muscle parameters and systemic muscle indices were analyzed using Spearman’s rank correlation and Bonferroni correction for multiple comparisons. Partial correlations controlling for sex and BMI and formal sex interaction tests were also performed. Group comparisons were conducted using independent t-tests or Mann–Whitney U tests.

**Results:**

The mean TMT was 7.6 ± 1.1 mm, whereas the mean TMA was 5.3 ± 1.4 cm². TMA demonstrated strong correlations with L3-CSA (*r* = 0.69, 95% CI = 0.52–0.81, *p* < 0.001, corrected *p* < 0.001), grip strength (*r* = 0.60, 95% CI = 0.39–0.75, *p* < 0.001, corrected *p* < 0.001), and waist circumference (*r* = 0.61, 95% CI = 0.40–0.76, *p* < 0.001, corrected *p* < 0.001). TMT showed moderate correlations with these same parameters: L3-CSA (*r* = 0.45, 95% CI = 0.22–0.63, *p* < 0.001, corrected *p* < 0.05); grip strength (*r* = 0.41, 95% CI = 0.18–0.60 *p* < 0.001, corrected *p* < 0.05), and waist circumference (*r* = 0.38, 95% CI = 0.18–0.60, *p* < 0.001, corrected *p* < 0.05). Neither TMT nor TMA correlated with gait speed or calf circumference. After partial correlation analyses that controlled sex and BMI, the correlations were substantially attenuated and none remained statistically significant. Sex-specific analyses revealed that men had significantly greater TMT and TMA compared to women (both *p* < 0.001). In multivariable linear regression models adjusting for BMI, a significant sex interaction was observed for the relationship between TMT and psoas muscle area (*β* = 0.79, 95% CI = 0.10–1.48, *p* for interaction = 0.025), indicating a stronger association in males. No other significant sex interactions were detected (all *p* > 0.05).

**Conclusions:**

In healthy young adults, MRI-derived TMT and, in particular, TMA are associated with key indicators of muscle mass and strength. TMT and TMA reflect global physiological status rather than serving as an independent functional indicator in this age group. TMA emerged as the more robust metric, warranting its prioritization in future research. Further validation in older adults and clinical populations with functional decline is essential to establish its clinical utility.

**Supplementary Information:**

The online version contains supplementary material available at 10.1186/s12891-026-09713-0.

## Introduction

Sarcopenia is a progressive, generalized skeletal muscle disorder characterized by the accelerated loss of muscle mass and function, commonly associated with aging and chronic diseases [1, 2]. Prevalence rates of sarcopenia are reported to range between 10% and 27% [3, 4]. This condition can lead to various adverse consequences, including impaired mobility, elevated morbidity and mortality, and a significant socioeconomic burden on both affected individuals and the public healthcare system [5, 6].

At present, there is no universally accepted diagnostic standard for sarcopenia [7, 8]. In Asian countries, most studies have adopted the 2019 diagnostic criteria established by the Asian Working Group for Sarcopenia (AWGS) [8]. According to these criteria, the diagnosis is based on three key components: muscle mass, muscle strength, and physical performance. Grip strength is commonly used to evaluate muscle strength [9]. Muscle mass is assessed using various techniques such as dual-energy X-ray absorptiometry (DXA), bioelectrical impedance analysis (BIA), computed tomography (CT), and magnetic resonance imaging (MRI), with the total skeletal muscle area at the level of third lumbar vertebra frequently quantified through CT and MRI [2, 10, 11]. Physical performance is typically measured using gait speed tests and chair stand tests [4]. However, conventional techniques for assessing muscle mass are often limited by high costs, potential radiation exposure, and limited availability in primary healthcare settings [12]. While MRI provides high‑resolution morphological assessment, its use in routine sarcopenia screening is limited by cost, availability, and the need for trained personnel. Therefore, temporal muscle measurements derived from MRI should be viewed as opportunistic or research‑based biomarkers rather than primary diagnostic tools in many care pathways. Furthermore, current assessment methods for sarcopenia face considerable challenges in specific patient populations, including those with stroke, where accurate evaluation of muscle strength, mass, and physical function is particularly difficult [1]. In addition, routine use of lumbar CT scans for sarcopenia diagnosis is uncommon among neurological or neurosurgical patients [13]. Consequently, there is an urgent need for novel, clinically practical, and easily applicable biomarkers for muscle health assessment.

Recently, there has been growing interest in TMT and TMA, which have been found to correlate with the risk of sarcopenia. Ham et al. suggested that TMT and TMA could serve as reliable surrogate markers for detecting low skeletal muscle mass in patients with cerebrovascular disease. However, their study did not examine the associations of TMT or TMA with preexisting muscle strength or functional assessments [14]. Similarly, Park et al. highlighted the potential of TMT as an indicator of sarcopenia, particularly in relation to muscle mass and strength among healthy adults. Their investigation, however, did not assess the associations between TMA and the key components of sarcopenia [15]. Additionally, TMT has been proposed as a practical tool for assessing sarcopenia risk and nutritional status in rehabilitation, particularly for stroke patients [16, 17]. However, the absence of healthy control groups in previous investigations substantially limits the generalizability of these findings. These findings suggest that TMT is an innovative and promising surrogate marker for sarcopenia. Compared to conventional assessment techniques, MRI-based TMT measurement offers advantages such as non-invasiveness, absence of ionizing radiation, and operational feasibility [15, 18]. The existing body of research, however, has notable limitations. Primarily, most studies have overlooked the inclusion of healthy controls and have focused exclusively on TMT, neglecting the potential role of TMA. Furthermore, the association between TMT and the multifaceted components of sarcopenia – muscle mass, strength, and physical performance remains incompletely explored from a multidimensional standpoint.

Therefore, this study aims to investigate the potential of TMT and TMA as indicators of systemic muscle by examining their associations with systemic muscle mass and strength in a healthy young cohort. This includes assessments of L3-CSA, grip strength, gait speed, and various anthropometric measurements.

## Methods

### Study design and participants

This single-center, prospective, cross-sectional study enrolled healthy adult volunteers who underwent brain and lumbar spine MRI at the First Affiliated Hospital of Shantou University Medical College between September and October 2025. The inclusion criteria were: (1) absence of neuromuscular diseases; (2) no severe diseases affecting muscle metabolism; and (3) provision of written informed consent. The exclusion criteria included: (1) contraindications to MRI; (2) current pregnancy or lactation; (3) history of major surgery or significant trauma within the previous month; and (4) regular use of medications known to affect muscle metabolism within the previous month. The study protocol was approved by the Ethics Committee of the First Affiliated Hospital of Shantou University Medical College (No. B-2025-177) and performed in accordance with the Declaration of Helsinki. Written informed consent was obtained from all participants, and all methods were carried out in accordance with relevant guidelines and regulations.

### MRI acquisition protocol

All MRI examinations were conducted using a 3.0 Tesla Siemens Trio MRI system. A single three-dimensional (3D) T1-weighted magnetization-prepared rapid gradient-echo (MPRAGE) sequence was acquired, encompassing the entire cranium. Key imaging parameters were as follows: repetition time/echo time (TR/TE) = 1600/2.52ms; flip angle = 9°; acquisition matrix = 256 × 256; field of view(FOV)= 256 × 256 mm²; slice thickness = 1.0 mm; isotropic voxel size = 1.0 × 1.0 × 1.0 mm^3^. TMT and TMA were quantified on the 3D T1-weighted images. In addition,2D fluid-attenuated inversion recovery (FLAIR) sequences were acquired for all participants to assess the presence or absence of structural brain lesions. For lumbar spine imaging, axial T2-weighted images (T2WI) were obtained using a Turbo Spin Echo (TSE) sequence. Key imaging parameters for the lumbar spine were as follows: TR/TE = 3300ms/100ms, Echo Train Length (ETL) = 28, flip angle = 137°, acquisition matrix = 320 × 320, FOV = 180 × 180 mm², slice thickness = 4 mm, and slice gap = 0.4 mm.

### Image analysis

Image analyses were conducted using the hospital’s picture archiving and communication system (PACS, GE Healthcare-Centricity RIS CE V3.0) by two radiologists, each with over 10 years of experience in musculoskeletal imaging. Both readers were blinded to the clinical information of the participants. To assess inter-observer agreement, the intraclass correlation coefficient (ICC) was calculated following all measurements.

### Measurement of TMT and TMA

**Fig. 1:**
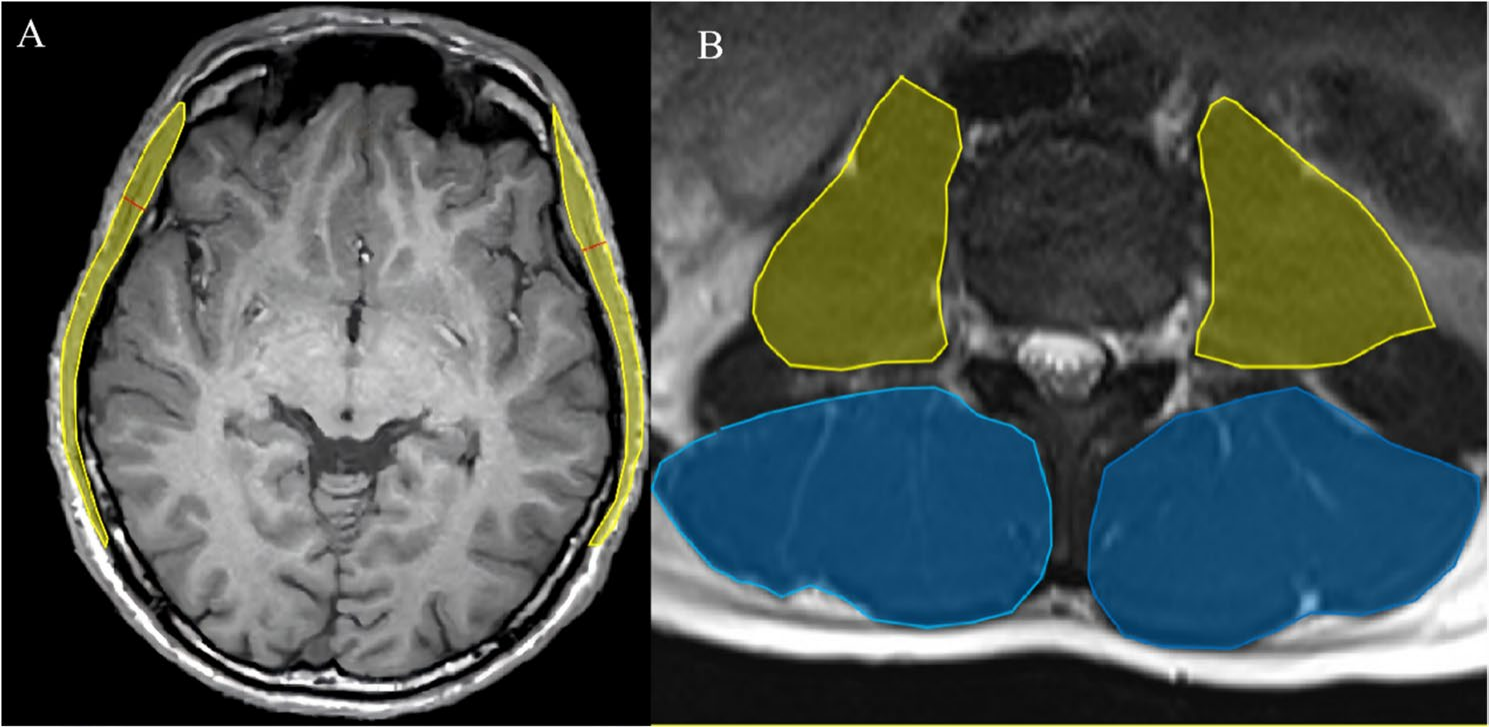
Measurement procedure for TMT, TMA and L3-CSA. **A** TMT was measured bilaterally, perpendicular to the muscle fibers, using the Sylvian fissure as the anterior-posterior anatomical reference; TMA was determined by manually tracing the outer contour of the temporal muscle on each side, and the average of both sides was calculated. **B **L3-CSA was obtained by manually tracing the outer contour of the lumbar muscle on each side

The evaluation of TMT and TMA was conducted using previously established protocols [11, 19]. TMT and TMA measurements were obtained at the slice corresponding to the orbital roof level on an axial plane, with the Sylvian fissure serving as the anatomical landmark for standardized positioning (Fig. 1). TMT was defined as the distance measured perpendicular to the long axis of the temporalis muscle, whereas TMA was calculated by manually delineating the muscle boundary on the same axial slice. Both parameters were assessed bilaterally, and the values from the left and right sides were averaged to derive the mean TMT and TMA for each participant.

### Measurement of L3-CSA

Axial T2-weighted images were used to select the slice that clearly depicted both transverse processes of the third lumbar vertebra [19]. On this specific slice, all relevant skeletal muscles, including the bilateral psoas and paraspinal muscles, were manually delineated to calculate the lumbar skeletal muscle cross-sectional area (CSA). Both the psoas and paraspinal muscle areas were assessed bilaterally, and the values from both sides were averaged to determine the mean psoas and paraspinal muscle areas for each participant. The cross-sectional areas of all segmented muscles were summed to obtain the total muscle area at the level of the third lumbar vertebra (Fig. 1), referred to as L3-CSA (cm²).

### Assessment of grip strength, gait speed (m/s), height (m), weight (kg), waist circumference (cm), hip circumference (cm), and calf circumference (cm)

Maximum grip strength (kg) of the dominant hand was measured using a standardized Jamar hydraulic hand dynamometer following the standardized protocol of the American Society of Hand Therapists. Participants were seated with their arms unsupported and the shoulder joints in a neutral position, and the highest value from three trials was recorded [20]. Usual gait speed (m/s) was assessed using a timed 6-meter walk test with 2-meter acceleration and deceleration zones to ensure steady-state walking following AWGS 2019 guidelines [8]. Timing was performed manually using a stopwatch, and the average of two trials was used for analysis. Anthropometric measurements included height (m), weight (kg), waist circumference (cm), hip circumference (cm), and maximum calf circumference (cm), all obtained using standardized procedures. Body mass index (BMI) was subsequently calculated as weight divided by height squared (kg/m²).

### Statistical analysis

Descriptive statistics were used to summarize continuous variables, which are presented as mean ± standard deviation. The normality of data distribution was assessed using the Shapiro-Wilk test. For between-group comparisons (e.g., male vs. female), independent-samples t-tests were applied to normally distributed data, while Mann-Whitney U tests were used for non-normally distributed data. Spearman correlation analysis was employed to examine the relationships between TMT, TMA, and systemic muscle indices, such as handgrip strength and L3-CSA. To account for multiple comparisons, we applied the Bonferroni correction, adjusting the significance threshold based on the number of comparisons performed. Subsequently, to evaluate the independent associations of TMT and TMA with muscle indices while controlling for potential confounders, we conducted partial correlation analyses adjusting for sex and BMI. To explore whether the relationship between temporal muscle morphology and systemic muscle health differed by sex, we performed multivariable linear regression analyses including interaction terms (Sex × TMT and Sex × TMA). Correlation strength was classified as weak (*r* < 0.4), moderate (0.4 ≤ *r* < 0.6), or strong (*r* ≥ 0.6). Intra- and inter-rater reliability of TMT and TMA measurements was assessed using the intraclass correlation coefficient (ICC) based on a single measure two-way mixed model. All statistical analyses were performed using IBM SPSS Statistics (version 26.0, Armonk, NY, USA) and R (version 4.3.2). *P*-values < 0.05 were considered statistically significant.

## Results

### Baseline characteristics and measurement reliability

The study comprised 61 healthy young adults, including 29 men (47.5%) and 32 women (52.5%), with a mean age of 21.7 ± 5.4 years. Baseline characteristics of the participants are detailed in Table 1. Measurement reliability was excellent, with intra- and inter-rater agreements for TMT and TMA showing intraclass correlation coefficients (ICC) of 0.90 and 0.91 for TMT, and 0.92 and 0.93 for TMA, respectively. The mean TMT was 7.6 ± 1.1 mm, while the mean TMA was 5.3 ± 1.4 cm². The mean L3-CSA was 51.9 ± 11.9 cm². The mean calf circumference was 34.7 ± 3.3 cm, the waist circumference was 74.2 ± 9.8 cm, and the hip circumference was 90.3 ± 12.8 cm. Additionally, the mean grip strength was 28.8 ± 6.9 kg, and the mean gait speed was 1.3 ± 0.2 m/s. As shown in Table 1, male participants exhibited significantly higher values than females for nearly all morphometric and functional measures. Specifically, males had larger TMA (6.1 ± 1.1 cm² vs. 4.4 ± 1.0 cm², *p* < 0.001), greater TMT (8.0 ± 1.1 mm vs. 7.2 ± 0.9 mm, *p* < 0.001), larger L3-CSA (65.6 ± 11.6 cm² vs. 40.1 ± 7.0 cm², *p* < 0.001), and stronger handgrip strength (34.2 ± 4.5 kg vs. 23.8 ± 4.5 kg, *p* < 0.001). Age and gait speed were not significantly different between males and females.

**Table 1:** Baseline characteristics of study participants stratified by sex

	Participants (*N* = 61)	Male (*N* = 29)	Female (*N* = 32)	*p*-value
Age, years	21.7 ± 5.4	25.3 ± 5.9	18.4 ± 0.8	<0.001 ‡
BMI (kg/m^2^)	21.5 ± 2.9	22.6 ± 2.5	20.5 ± 2.9	0.005 ‡
Calf circumference(cm)	34.7 ± 3.3	35.1 ± 3.5	34.4 ± 3.2	0.43 ‡
Hip circumference(cm)	90.3 ± 12.8	92.2 ± 8.0	88.6 ± 15.9	0.347†
Waist circumference(cm)	74.2 ± 9.8	80.6 ± 8.6	68.4 ± 6.8	<0.001 ‡
Grip strength(kg)	28.8 ± 6.9	34.2 ± 4.5	23.8 ± 4.5	<0.001 ‡
Gait speed(m/s)	1.3 ± 0.2	1.3 ± 0.2	1.3 ± 0.2	0.69†
TMT(mm)	7.6 ± 1.1	8.0 ± 1.1	7.2 ± 0.9	<0.001 ‡
TMA(cm^2^)	5.3 ± 1.4	6.1 ± 1.1	4.4 ± 1.0	<0.001 ‡
Psoas muscle area(cm^2^)	7.9 ± 2.6	10.2 ± 2.0	5.9 ± 0.9	<0.001†
Paraspinal muscle area(cm^2^)	18.0 ± 5.6	22.5 ± 4.4	14.0 ± 2.8	<0.001†
L3-CSA(cm^2^)	51.9 ± 15.9	65.6 ± 11.6	40.1 ± 7.0	<0.001†

†*P* < 0.05 by Mann–Whitney U test, ‡*P* < 0.05 by t-test

The data are presented as mean ± standard deviation

*Abbreviations*: *BMI* Body mass index, *TMT* Temporal muscle thickness, *TMA* Temporal muscle area, *L3-CSA* Lumbar skeletal muscle cross-sectional area at the third lumbar vertebra

### Correlations of temporal muscle parameters with systemic muscle indices

**Fig. 2:**
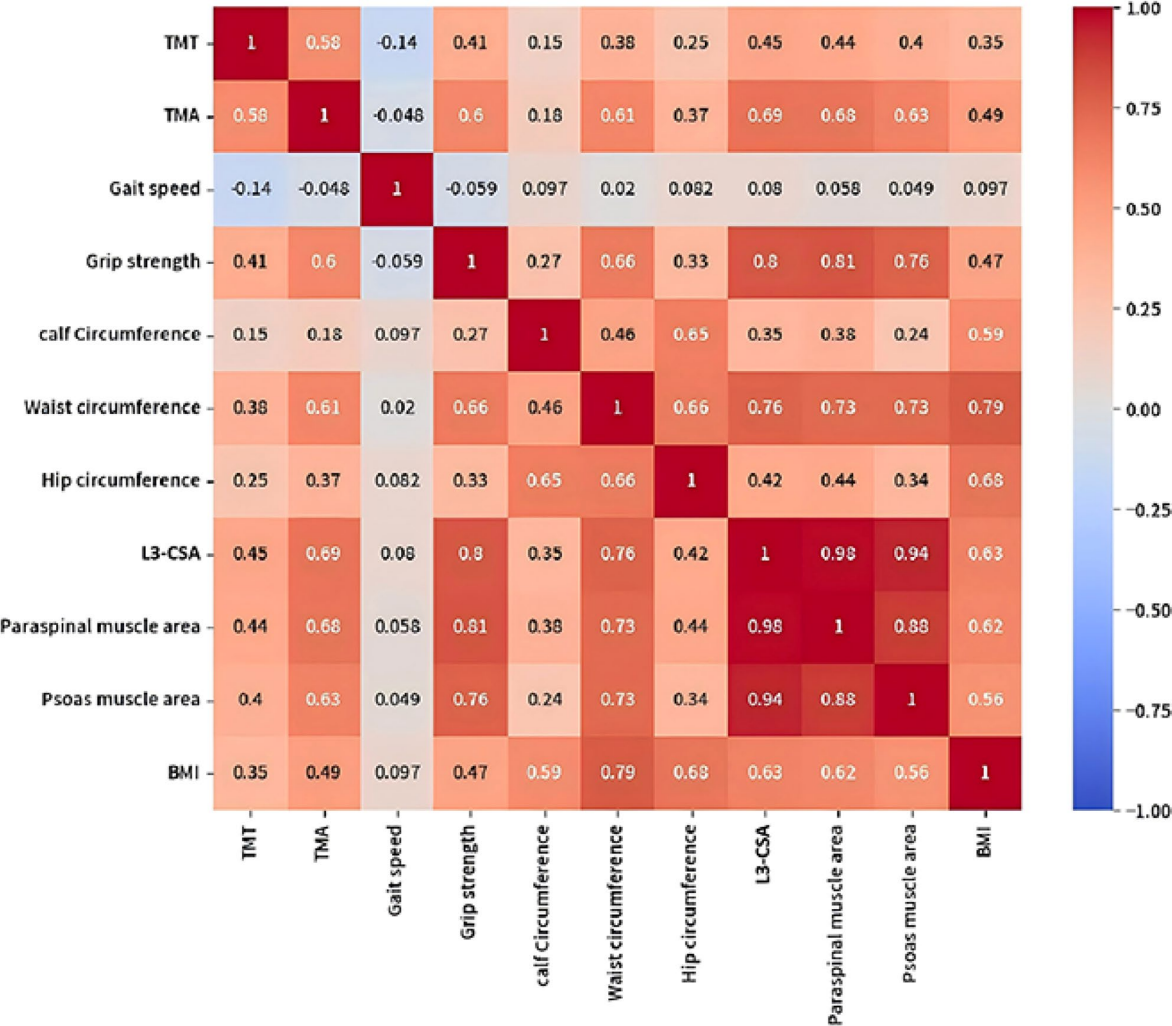
Heatmap of Spearman correlation matrix among temporal muscle parameters and systemic muscle indices. Positive correlations are shown in red, and negative correlations in blue, with color intensity proportional to the correlation strength

After applying the Bonferroni correction to adjust for multiple testing, the correlations between TMA/TMT and L3-CSA, grip strength, and waist circumference remained statistically significant, confirming the robustness of these findings. As shown in Fig. 2 TMA demonstrated strong correlations with L3-CSA (*r* = 0.69, 95% CI = 0.52–0.81, *p* < 0.001, corrected *p* < 0.001), grip strength (*r* = 0.60, 95% CI = 0.39–0.75, *p* < 0.001, corrected *p* < 0.001), and waist circumference (*r* = 0.61, 95% CI = 0.40–0.76, *p* < 0.001, corrected *p* < 0.001). In contrast, TMT showed moderate correlations with these same parameters: L3-CSA (*r* = 0.45, 95% CI = 0.22–0.63, *p* < 0.001, corrected *p* < 0.05); grip strength (*r* = 0.41, 95% CI = 0.18–0.60 *p* < 0.001, corrected *p* < 0.05), and waist circumference (*r* = 0.38, 95% CI = 0.18–0.60, *p* < 0.001, corrected *p* < 0.05). In comparison, neither TMA nor TMT showed significant correlations with gait speed or calf circumference. After correction, all non-significant findings in the raw analysis remained non-significant after correction.

**Fig. 3:**
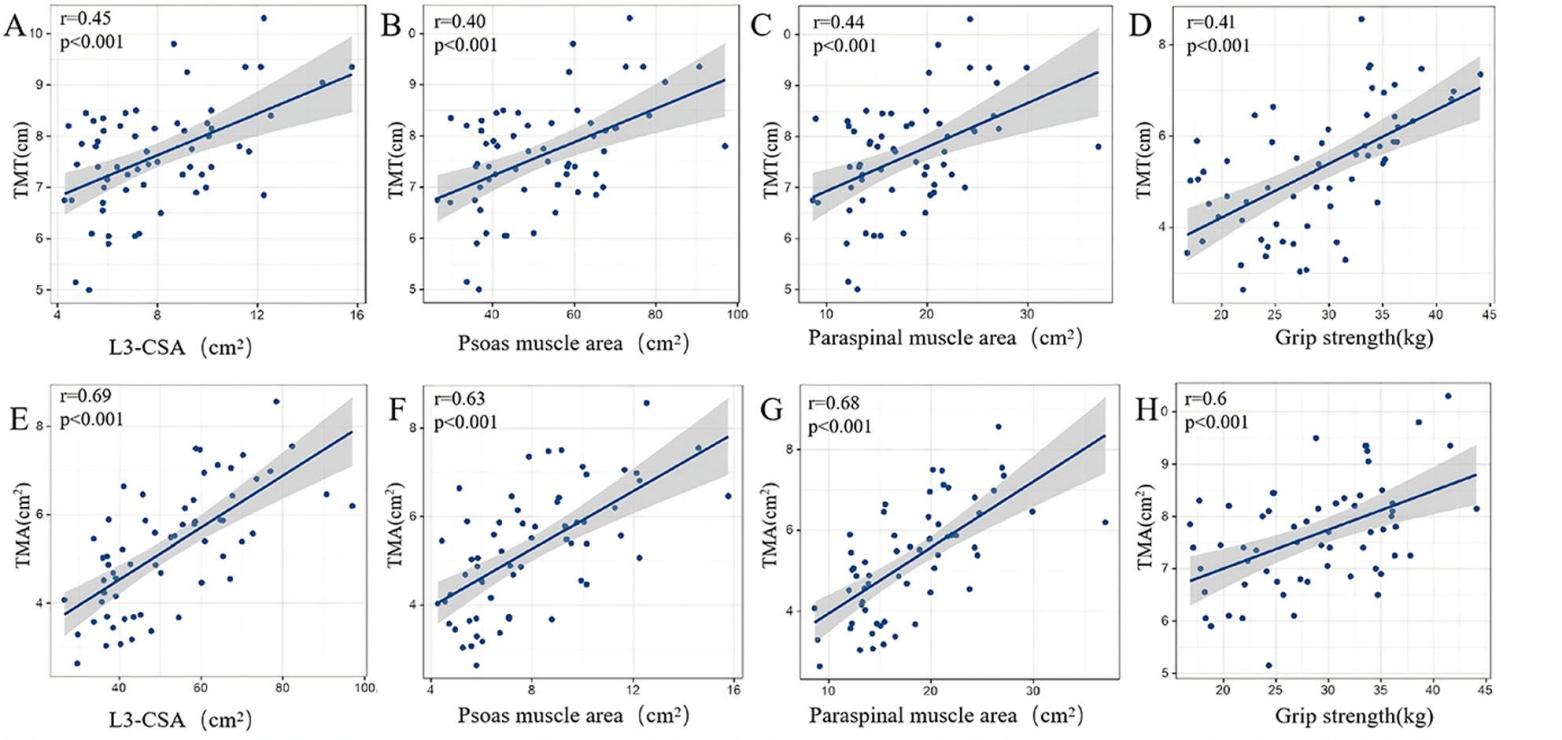
Correlations of TMT/TMA with lumbar muscle CSA and grip strength. Scatter plots of (**A**, **E**) TMT/TMA and L3-CSA; **B**, **F**) TMT/TMA and psoas muscle area; **C**,** G **TMT/TMA and paraspinal muscle area; **D**, **H **TMT/TMA and grip strength

Partial correlation analyses were performed to examine the independent associations of TMT and TMA with systemic muscle indices after controlling for sex and BMI (Table 2). Following adjustment, the correlations were substantially attenuated and none remained statistically significant. These results indicate that the unadjusted correlations between TMT, TMA morphology and systemic muscle indices were largely explained by sex differences and BMI. Figure 3 shows the linear associations of TMT and TMA with L3-CSA, and grip strength. As lumbar muscle area and grip strength increase, both TMT and TMA also increase, suggesting a positive association between cranial and systemic muscle morphology. Sex-specific analyses revealed that correlation coefficients for TMT and TMA with both L3-CSA and grip strength were higher in males than in females (Fig. S1). In multivariable linear regression models adjusting for BMI, a significant sex interaction was observed for the relationship between TMT and psoas muscle area (*β* = 0.79, 95% CI = 0.10–1.48, *p* for interaction = 0.025), indicating that the positive association was significantly stronger in males than in females. Marginally significant interactions were found for TMT with L3-CSA (*β* = 3.61, 95% CI = -0.07-7.29, *p* = 0.055) and TMA with grip strength (*β* = 2.01, 95% CI = -0.07-4.09, *p* = 0.059). No other significant sex interactions were detected (all *p* > 0.05).

**Table 2:** Partial correlation between TMT and TMA with systemic muscle indices after controlling for sex and BMI

	TMT		TMA	
	Partial *r*	*p*-value	Partial *r*	*p*-value
Calf Circumference	-0.03	0.82	-0.05	0.69
Waist circumference	0.06	0.66	0.11	0.41
Hip circumference	0.07	0.58	0.21	0.11
Grip strength	0.21	0.16	0.14	0.30
Gait speed	-0.19	0.15	-0.13	0.33
Psoas muscle area	0.14	0.28	0.06	0.67
Paraspinal muscle area	0.21	0.10	0.24	0.07
Lumbar muscle area	0.24	0.06	0.22	0.09

^*^ *p*-value of < 0.05 was considered to indicate statistical significance

## Discussion

This study demonstrates that MRI-derived TMT and TMA are significantly correlated with key indicators of muscle mass and strength in a cohort of healthy young adults. TMA reflects global physiological status rather than serving as an independent functional indicator in this age group. Notably, TMA demonstrated stronger correlations than TMT. These findings extend previous research conducted in elderly and clinical populations to a younger cohort [19, 21]. To our knowledge, this is the first study to examine the correlation between TMT, TMA, and L3-CSA in a healthy cohort, providing fundamental reference data and suggesting the potential of the temporal muscle as an indicator of systemic muscle mass and strength.

Previous literature has reported that TMT and TMA increase from birth to a peak at approximately 30–40 years of age, followed by a subsequent decline. Moreover, males tend to have relatively higher TMT and TMA compared to females [13]. In our study population, similar gender differences in TMT and TMA were observed. These differences can be attributed to intrinsic sex-specific differences in body composition, baseline muscle mass, and hormonal influences, particularly the anabolic effects of testosterone [22].

In this study, both TMT and TMA exhibited moderate-to-strong correlations with L3-CSA and handgrip strength. These findings are consistent with previous reports. For example, Pesonen et al. [13]. demonstrated that temporal muscle morphology can serve as a reliable surrogate marker for systemic muscle status, particularly in populations where lumbar imaging is unavailable. Similarly, Park et al. [23]. reported that TMT correlated with grip strength in acute stroke patients, further supporting its role as a practical indicator of overall muscle function. These findings establish baseline associations between temporal muscle metrics and systemic muscle mass in healthy young adults, which may serve as a foundation for future longitudinal studies investigating how these relationships change with aging. The assessment of TMT and TMA can be seamlessly integrated into standard brain CT or MRI examinations, offering significant and valuable advantages in the diagnostic process.

Our results indicate that TMA has a stronger correlation with L3-CSA (*r* = 0.69) and handgrip strength (*r* = 0.60) compared to TMT. We hypothesize that TMA, by encompassing the entire semi-axial cross-sectional region, is less susceptible to measurement variability than TMT, which may be more affected by minor differences in defining measurement boundaries. Although TMT is more extensively documented in the literature [11, 18], TMA has consistently demonstrated a significant association with adverse clinical outcomes in conditions such as glioblastoma and subarachnoid hemorrhage [14, 24, 25]. TMA may also be more compatible with automated segmentation approaches than TMT. Consequently, TMA represents a more robust and promising metric, pending validation for future research and clinical integration.

Notably, the partial correlation analyses revealed a pivotal finding: substantial attenuation of the correlations between TMT, TMA and systemic muscle indices after adjusting for sex and BMI. These findings suggest that the unadjusted associations between temporal muscle morphology and systemic muscle indices are driven largely by sex and BMI, rather than being independent. This observation is consistent with previous literature. First, Katsuki et al. [26] showed in a large Japanese cohort (*n* = 360) that TMT correlates with weight and BMI in both men and women, indicating that nutritional status (reflected by BMI) is a common determinant of cranial and systemic muscle size. Second, a systematic review by Engman et al. [27] synthesized evidence that intramuscular sex hormone concentrations positively correlate with muscle mass and strength in males and are regulated, at least in part, independently of circulating hormones, as well as modulated by aging and physical activity. Together, these findings suggest that TMT and TMA reflect global physiological status rather than serving as independent muscle-specific predictors, capturing the combined influence of sex hormones and nutritional state. These findings highlight the need to consider these confounders when using temporal muscle morphology as a biomarker in future studies.

Our analysis reveals that temporal muscle parameters were not associated with functional measures. The lack of correlation with gait speed suggests that MRI‑derived morphological indicators do not capture the functional dimension of muscle health in young, functionally preserved individuals. This aligns with the null association between TMT and the five-times-sit-to-stand test reported by Park et al. in a similarly healthy cohort [15]. The uniformly high gait speed in our cohort (1.3 ± 0.16 m/s) likely contributed to this lack of correlation, creating a restricted range and ceiling effect. In contrast, gait speed is a well-established marker of sarcopenia severity in older or clinically compromised populations, where functional decline is more pronounced [28]. Thus, in young healthy individuals, temporal muscle morphology is associated with muscle mass but not with the functional dimension of muscle health. This underscores that MRI-derived morphological indicators should not be equated with functional status, particularly in populations with preserved muscle function. Future studies in older or clinical cohorts with greater functional heterogeneity are needed to determine whether temporal muscle morphology captures functional associations in those settings.

Our results reveal a significant sex interaction in the relationship between TMT and psoas muscle area (*p* for interaction < 0.05), indicating that the association between cranial and axial skeletal muscle morphology is stronger in males. This finding may reflect sex-related differences in muscle fiber composition or hormonal influences on muscle maintenance. Foundational work by Ranganathan et al. [29] reported a robust correlation between TMT and total psoas area (*r* = 0.57), supporting TMT as a surrogate marker of systemic muscle health. Our study extends this by showing that the association is further modified by sex. Notably, the lack of significant sex interactions in the associations between temporal muscle parameters and other measures of systemic muscle health suggests that cranial muscle metrics are generalizable across sexes. Consistent with this, Han et al. [14] found that TMT exhibits significant correlations with skeletal muscle mass independent of sex (male: *r* = 0.520; female: *r* = 0.706). These observations are in line with the life-course framework for muscle health promotion emphasized in the AWGS 2025 consensus [30]. Taken together, our findings support TMT as a clinically accessible indicator of systemic muscle status in both men and women.

This study has several limitations. First, the sample size was small (*n* = 61) and drawn from a single institution, which may limit the generalizability of the findings. Second, although L3-CSA is a widely accepted surrogate for whole-body muscle mass, it does not provide a direct assessment of muscle mass. Third, our study population comprised young, healthy adults, whereas sarcopenia primarily affects older or clinically vulnerable individuals, limiting the generalizability of our findings to those at highest risk. Fourth, the lack of association with functional measures (gait speed) is a notable limitation. Although possibly due to a ceiling effect in healthy young adults, this finding confirms that morphological indicators alone do not reflect functional muscle health. Fifth, while the Bonferroni correction controlled for Type I errors, it may have concurrently increased the risk of Type II errors, potentially obscuring clinically meaningful associations that did not survive the adjusted threshold. To address these limitations, future multicenter, longitudinal studies are needed to confirm causal relationships, establish standardized imaging protocols, and generate age- and sex-stratified reference values to validate the predictive utility of TMT and TMA.

## Conclusion

This study demonstrates that in healthy young adults, MRI-derived temporal muscle morphology correlates with systemic muscle mass and strength. TMT and TMA primarily reflect global physiological status rather than serving as independent functional indicators in this age group. Compared to TMT, TMA emerged as the more robust and reliable metric and should be prioritized in future research. These findings support the feasibility of using temporal muscle morphology as an indicator of muscle health. However, further validation in older adults and patient populations with functional decline is essential to establish its clinical utility.

## Supplementary Material

Supplementary Material 1. Figure S1. Correlations of TMT/TMA with lumbar muscle CSA and grip strength stratified by sex in healthy young adults: females are represented in blue and males in red. Scatter plots of (A, E) TMT/TMA and L3-CSA; (B, F) TMT/TMA and psoas muscle area; (C, G) TMT/TMA and paraspinal muscle area; (D, H) TMT/TMA and grip strength.

## Data Availability

The datasets generated and analyzed during this study are available from the corresponding author upon reasonable request.

## Abbreviations

TMT: Temporal muscle thickness
TMA: Temporal muscle area
DXA: Dual-energy X-ray absorptiometry
BIA: Bioelectrical impedance analysis
CT: Computed tomography
MRI: Magnetic resonance imaging
BMI: Body mass index
AWGS: Asian Working Group for Sarcopenia
CSA: Cross-sectional area
