# Supplementary material for: Association between temporal muscle morphology based on MRI and muscle mass and strength in healthy young adults: a cross-sectional study
Source: BMC Musculoskelet Disord. 2026 Mar 7;27:312. doi: 10.1186/s12891-026-09713-0 (PMC13081373; doi:10.1186/s12891-026-09713-0)
Supplement: Supplementary file 1 — Supplementary Material 1. Figure S1. Correlations of TMT/TMA with lumbar muscle CSA and grip strength stratified by sex in healthy young adults: females are represented in blue and males in red. Scatter plots of (A, E) TMT/TMA and L3-CSA; (B, F) TMT/TMA and psoas muscle area; (C, G) TMT/TMA and paraspinal muscle area; (D, H) TMT/TMA and grip strength. [file 12891_2026_9713_MOESM1_ESM.docx]

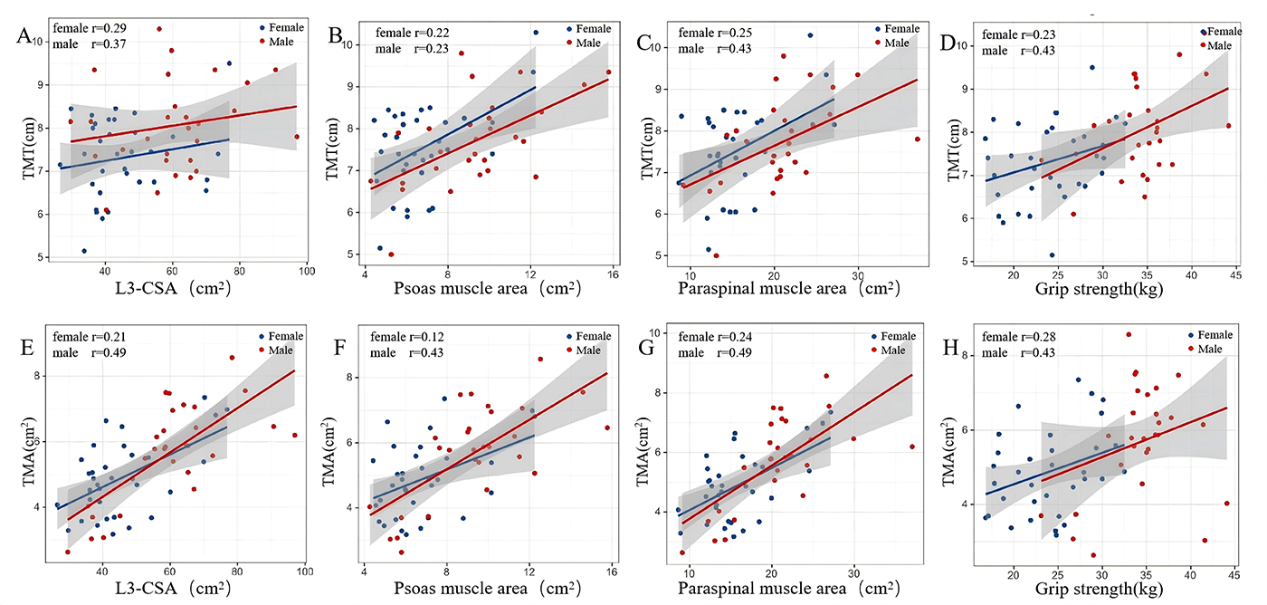
 Supplementary Material 1. Figure S1. Correlations of TMT/TMA with lumbar muscle CSA and grip strength stratified by sex in healthy young adults: females are represented in blue and males in red. Scatter plots of (A, E) TMT/TMA and L3-CSA; (B, F) TMT/TMA and psoas muscle area; (C, G) TMT/TMA and paraspinal muscle area; (D, H) TMT/TMA and grip strength.
